# Callosal abnormalities, altered cortisol levels, and neurocognitive deficits associated with early maltreatment among adolescents: A voxel‐based diffusion‐tensor imaging study

**DOI:** 10.1002/brb3.2009

**Published:** 2021-01-15

**Authors:** Paulo Jannuzzi Cunha, Fabio L.S. Duran, Paula Approbato de Oliveira, Tiffany M. Chaim‐Avancini, Ana Luiza V. Milioni, Mariella Ometto, Paula Squarzoni, Pedro P. Santos, Sheila C. Caetano, Geraldo F. Busatto, Sandra Scivoletto

**Affiliations:** ^1^ Laboratory of Psychiatric Neuroimaging (LIM‐21) Instituto de Psiquiatria, Hospital das Clinicas HCFMUSP Faculdade de Medicina Universidade de Sao Paulo Sao Paulo Brazil; ^2^ Departamento e Instituto de Psiquiatria Hospital das Clinicas HCFMUSP Faculdade de Medicina Universidade de Sao Paulo Sao Paulo Brazil; ^3^ Departamento de Psiquiatria Universidade Federal de São Paulo (UNIFESP) São Paulo Brazil

**Keywords:** corpus callosum, diffusion‐tensor imaging, emotional abuse

## Abstract

**Introduction:**

Neuroimaging studies have shown callosal abnormalities among maltreated subjects, but little is known about the functional and neurobiological correlates of these supposed developmental alterations. The aim of this study was to investigate childhood maltreatment (CM), neurocognitive functioning, cortisol levels, and corpus callosum (CC) integrity among adolescents.

**Methods:**

One hundred and seven subjects underwent magnetic resonance imaging (MRI) with voxel‐based diffusion‐tensor imaging (DTI) and the Crossed Finger Localization Test (CFLT). Psychopathology was investigated with the Schedule for Affective Disorders and Schizophrenia (K‐SADS‐PL); CM was detailed by the Childhood Trauma Questionnaire (CTQ), and salivary cortisol levels were measured by immunoassay.

**Results:**

Higher levels of CM were associated with current lower CFLT scores, mainly in the CROSSED condition, involving interhemispheric communication of sensorimotor information (*p* < .05) and with reduced fractional anisotropy (FA) in the *splenium* of the CC (*p* < .01). Deficits in the CFLT were also associated with higher cortisol levels (*p* < .05).

**Conclusion:**

The association among CM, neuropsychological abnormalities, callosal microstructure alterations, and cortisol levels suggests an altered pattern of brain interhemispheric connectivity among maltreated adolescents. Further studies are needed to investigate the extent to which these sensorimotor deficits and abnormal cortisol levels may be possible mediators of negative neurodevelopmental trajectories and adult psychopathology.

## Graphical abstract

In our study, objective measures of childhood maltreatment were associated with abnormal white matter microstructure in posterior regions of the CC (splenium), and correlated with specific neurocognitive impairments and abnormal cortisol levels.

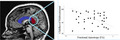

## 1 INTRODUCTION

Childhood maltreatment (CM) has been associated with brain structural and functional abnormalities (De Bellis, 2001; De Bellis et al., 2015; Hart et al., 2018; Lupien et al., 2009; Puetz et al., 2017; Tendolkar et al., 2018). More recently, diffusion‐tensor imaging (DTI) has emerged as an important MRI‐based technique for studying the white matter (WM) brain microstructure, by providing measures of fiber myelination and organization, such as fractional anisotropy (FA) (Le Bihan, 2003; Mori, 2007; Mori et al., 2005). One of the most common findings among maltreated subjects has been a significant decreased FA in the corpus callosum (CC), an important WM commissure linking the left and right hemispheres (De Bellis et al., 2015; Huang et al., 2012; Jackowski et al., 2008; Lee et al., 2018; Lim et al., 2020; McCarthy‐Jones et al., 2018; Seckfort et al., 2008). The rationale behind these findings has been associated with a chronic disruptive effect of repeated high doses of cortisol on the normal processes of neural development and maturation during early childhood (Lim et al., 2020). Acute stress can cause significant and transient alterations in cortisol release (Bunea et al., 2017; Lupien et al., 2009), which chronically and/or particularly during certain critical periods of neurodevelopment can significantly interfere with hypothalamic–pituitary–adrenal (HPA) system causing long‐lasting effects on myelination and integrity of WM tracts (Howell et al., 2013). Then, the hormone cortisol has been considered a biological marker of long‐term effects of early‐life adversity (Bunea et al., 2017). However, the study of WM tracts connecting brain regions implicated in childhood maltreatment has received only little attention; then, WM studies with DTI linking CC microstructural abnormalities to cortisol are still lacking. Moreover, to the best of our knowledge, no imaging study to date has directly demonstrated a functional or neuropsychological correlate of the supposed callosal abnormalities among maltreated subjects, so the clinical relevance of such abnormalities remains largely unknown (McCarthy‐Jones et al., 2018). The Crossed Finger Localization Task (CFLT) is a neuropsychological test that requires interhemispheric transfer of sensorimotor (tactile) information across the CC (Rushe et al., 2007; Satomi et al., 1991). Although CFLT has been considered a validated test of callosal functioning (Chaim et al., 2010; Geffen et al., 1985; Volpe et al., 1982), it has never been used to evaluate individuals with a history of maltreatment. To understand the impact of early‐life adversities on sensorimotor integration could be of great value in the context of maltreated children due to implications with affective processing and personality traits.

The aims of the study were to investigate WM microstructural integrity of the CC in a sample of maltreated adolescents using DTI, and also to study possible associations between FA alterations in separate callosal areas (anterior, body, and splenium) with CC functioning and cortisol levels. We hypothesized that adolescents with a history of CM would present with reduced WM integrity in the CC, cortisol alterations, and deficits in interhemispheric communication.

## 2 METHODS

### 2.1 Participants

Subjects were included in the group of maltreated adolescents (MA) if they presented a history of significant childhood maltreatment and are being under medical or psychological treatment for maltreatment as described elsewhere (Ometto et al., 2016). The comparison adolescent (CA) group was composed of youths with a similar socioeconomic background (when compared to MA) and without a history of maltreatment. CA were recruited in a multidisciplinary service that offers a variety of activities including psychosocial and medical interventions for low‐income families. Exclusion criteria for all subjects were as follows: history of neurological disorders, autism, substance use disorder (SUD), psychosis, and traumatic brain injury (TBI). Of a total of 107 adolescents, 12 were excluded due to imaging artifacts and silent nonexpected brain findings, so the following 95 individuals were divided in 58 MA and 37 CA.

### 2.2 Instruments and examinations

#### 2.2.1 Psychiatric Symptoms and Childhood Maltreatment (CM)

Psychiatric symptoms were evaluated with the *Schedule for Affective Disorders and Schizophrenia for School‐Age Children—Present and Lifetime version* (K‐SADS‐PL), which demonstrates very good validity and reliability—the interrater agreement in scoring and diagnosis range between 93% and 100%, and the test–retest reliability (kappa) coefficients were 0.77–1.00 for present and/or lifetime diagnoses (Kaufman et al., 1997). The *Childhood Trauma Questionnaire* (CTQ‐SF)—total score—was used to assess different levels of exposure to childhood maltreatment (Bernstein et al., 2003; Grassi‐Oliveira et al., 2006), in order to analyze how severity of childhood maltreatment experiences could correlate with later WM alterations, cortisol levels, and cognitive results. The CTQ‐SF has also good validity and reliability coefficient scores. The interrater reliability ranges from 0.79 to 0.94, and the test–retest coefficient was 0.88 (Bernstein et al., 1994). The history of moderate to severe and extreme childhood maltreatment was identified using the cutoff proposed by Bernstein et al. (2003)—percentile 90.

#### 2.2.2 Callosal function examination

Callosal function was assessed with the *Crossed Finger Localization Test (CFLT)* (Rushe et al., 2007; Satomi et al., 1991). In this task, a fingertip on the one hand is touched by the experimenter out of the view of the subject; he or she must then identify which finger was touched either on the same hand (SAME condition) or on the opposite hand (CROSSED condition), by bringing the thumb of that hand to touch the appropriate finger. Only the CROSSED condition demands cross‐callosal transfer of tactile information (Rushe et al., 2007). Two series of trials are carried out for each condition, initially with only one finger being touched at a time, and subsequently with two fingers touched consecutively. One point is given to each correct response, giving a maximum total score of 32 points for each condition.

#### 2.2.3 Salivary Cortisol

Saliva samples were collected from adolescents in the morning, before the assessment. Saliva samples were obtained by placing cotton rolls in the adolescents´ mouth, until cotton was completely saturated. Cotton rolls were then placed in the saliva‐collecting devices (Salivette^®^). The samples were then centrifuged and immediately frozen for later analysis. Cortisol levels were determined using an immunoassay (IA) kit.

#### 2.2.4 Pubertal Status

Pubertal status was evaluated by the Pubertal Developmental Scale (PDS), which is a short self‐report and noninvasive measure based on five markers of pubertal development, including physical aspects such as growth of body hair, skin changes (e.g., pimples), voice changes/facial hair (for boys), breast development and menstruation (for girls), and self‐perception about his or her puberty status (Petersen et al., 1988). The PBS has adequate validity and reliability. The internal consistency ranges between 0.91 and 0.96 and the test–retest reliability was 0.81–0.92 (Koopman‐Verhoeff et al., 2020). The PBS was used for classifying each subject from MA and CA in two groups: pre‐, early‐, and midpuberty (for boys—PBS = 3–8; for girls—PBS = 2–3 and no menarche) versus late‐ and postpuberty (for boys—PBS: 9–12; for girls—PBS = 7–8 and menarche).

### 2.3 Neuroimaging

#### 2.3.1 DTI acquisition

A T1‐weighted magnetization‐prepared gradient echo sequence (MPRAGE) acquisition was performed using a 1.5T MRI SIEMENS Espree scanner (Siemens) including the following parameters: TR = 2,400 ms, TE = 3.65 ms, NEX = 1, field of view (FOV) = 240 mm, slice thickness = 1.2 mm (no gap between slices), slices = 160, flip angle = 8°, matrix = 192 × 192 pixels, voxel size = 1.3 × 1.3 × 1.2 mm. The DTI sequence was synchronized to the cardiac gating and consisted of one T2‐weighted image without diffusion gradient (*b* = 0 s/mm^2^) plus diffusion‐weighted images (DWI) acquired along 64 noncollinear directions (*b* = 1,000 s/mm^2^). DWI was based on an echo‐planar image (EPI) acquisition with the following parameters: TR = 8,000 ms, TE = 110 ms, NEX = 2, FOV = 240 mm, slice thickness = 2.7 mm (no gap between slices), 50 slices, matrix = 120 × 120 pixels. The two sequences were acquired in 25 min. Individual image inspection was performed by an expert neuroradiologist aiming to identify possible silent brain lesions and artifacts that could interfere with image processing and analysis.

#### 2.3.2 Processing of neuroimaging data

The diffusion‐tensor images were analyzed using FMRIB Software Library (FSL) version 5.0.6 (http://fsl.fmrib.ox.ac.uk/fsl/fslwiki/FSL) (Andersson et al., 2007). The distortions caused by the large diffusion‐sensitizing gradients and motion were corrected, using as a guide the *b*0 volume. Afterward, an automated procedure for segmenting and making brain and nonbrain tissues was applied; then, eigenvalues and eigenvectors were estimated in order to compute FA values, which were used in the present study. Then, we used Tract‐Based Spatial Statistics (TBSS) on the FSL to process fiber tracts. TBSS involves several preprocessing steps before final analyses. First, FA maps of all subjects were aligned to each other based on free‐form deformations and B‐spline nonlinear registration approach (Rueckert et al., 1999). The target image was then affine‐aligned into MNI standard space, and every image was transformed into 1 × 1 × 1 mm3 MNI standard space by combining the obtained linear and nonlinear transformation parameters. Next, the mean FA image was computed and thinned to create a mean FA (*skeletonization*). Then, each subject's aligned FA data were then projected onto this skeleton. Such procedure is taken by searching the highest FA values of the subject's perpendicular tract direction within mean FA skeleton. The data were then exported to the *Statistic Parametric Mapping* (SPM) program, version 8.0 (https://www.fil.ion.ucl.ac.uk/spm/), to conduct the between‐groups comparative statistical analysis. For correlation analyses (i.e., CTQ vs. FA values), we extracted individual FA values for each subregion of the CC also using the SPM.

### 2.4 Statistical analysis

Sociodemographic characteristics and clinical data were compared between MA and CA using Student's *t* tests for continuous variables with normal distribution, and with the Mann–Whitney tests when the distribution was not normal. The chi‐square tests were used to compare categorical variables (i.e., sex, and ethnicity). Voxel‐based comparisons of FA values were performed using SPM 8.0. FA values were standardized to the global mean using proportional scaling, thus controlling for interindividual differences in FA. First, we inspected for the presence of FA alterations in the MA group in the main CC areas using an automated atlas of WM for DTI from researchers at Johns Hopkins University (JHU) (Mori, 2007), including the three main subdivisions of the corpus callosum (CC): genu, body (medium part), and splenium (posterior subarea) of the CC. Correlated analyses including CTQ, CFLT, cortisol levels, and FA values were performed using the Spearman or Pearson correlation coefficients, depending on the distribution of data, using the Statistical Package for the Social Sciences (SPSS), version 17.0. For all comparisons and correlations, *p* ≤ .05 was considered statistically significant, and results from DTI comparative analyses were reported only if surviving a FWE‐correction threshold (correction for multiple comparisons).

## 3 RESULTS

### 3.1 Sociodemographic characteristics, psychiatric comorbidity, and measures of childhood maltreatment

The samples (MA vs. CA) did not differ significantly in terms of age, gender, education (in years), laterality, ethnicity, pubertal status, impulsivity, anxiety, and cortisol levels (*p* > .05; see Table 1). However, we found higher frequencies of psychiatric comorbidity among MA, including a higher prevalence of depressive, conduct, and attention‐deficit/hyperactivity disorder (ADHD), when compared to CA (*p* < .01).

**Table 1:** Sociodemographic data, psychiatric comorbid disorders, and measures of childhood maltreatment among maltreated (MA) and comparison adolescents (CA)

	MA (*n* = 58)	CA (*n* = 37)	*p*
Age (years)	13.03 (±1.21)	12.65 (±1.03)	.113^χ^
Sex (boys/girls)	33/25	22/15	.805^β^
Education (years)	7.28 (±1.29)	7.43 (±0.98)	.532^χ^
Laterality			
Right‐handed	52 (94.5%)	34 (91.9%)	.681
Left‐handed	3 (5.5%)	3 (8.1%)	
Ethnicity			
White	19 (32.8%)	21 (56.8%)	.069^β^
Black	31 (53.4%)	13 (35.1%)	
Others	8 (13.8%)	3 (8.1%)	
Pubertal status (PDS)			
Pre‐, early‐, and midpuberty	32 (57.1%)	22 (59.5%)	.834
Late‐ and postpuberty	24 (42.9%)	15 (40.5%)	
Cortisol levels			
Cortisol level	0.21 (±0.14)	0.26 (±0.18)	.277
Comorbid psychiatric disorders			
Anxiety disorders (other than PTSD)^a^	7 (12.1%)	3 (8.1%)	.542
Depressive disorders	18 (31%)	0 (0%)	<.01**
Conduct disorders	11 (19%)	0 (0%)	<.01**
ADHD	20 (34.5%)	3 (8.1%)	<.01**
Impulsivity (BIS)	69.04 (±12.97)	66.46 (±8.73)	.306
Childhood maltreatment (CTQ)	45.33 (±18.27)	36.50 (±10.60)	.004**^,χ^

Note

Age and education are presented in means and standard deviations: *M* (±*SD*). Sex, ethnicity, and psychiatric disorders are presented in absolute frequency. Level of statistical significance *p ≤ .05; **p < .01; *p* ^β^ = Chi‐square test; *p* ^χ^ = *Student's* *t* test.

Abbreviations: ADHD, attention‐deficit/hyperactivity disorder; BIS, Barrat Impulsivity Scale; CA, comparison adolescents; CTQ, Childhood Trauma Questionnaire; MA, maltreated adolescents; PDS, Pubertal Developmental Scale; PTSD, post‐traumatic stress disorder.

^a^ Anxiety disorders include panic disorder, generalized anxiety disorder, phobia‐related disorders.

### 3.2 Callosal functioning (CFLT)

The MA and CA groups differed significantly in their performance on the CFLT—SAME and CROSSED conditions (see Table 2). Alterations on CFLT were more frequent on the CROSSED conditions. Among MA, CFLT scores were lower on the one‐fingered CROSSED (right‐hand, *p* = .02) and SAME (left‐hand, *p* < .05) conditions, when directly compared to CA. Also, MA had impaired CFLT performance on the two‐fingered CROSSED conditions (for both right and left hands, *p* = .01), relative to CA.

**Table 2:** Performance on the CFLT in maltreated (MA) and comparison adolescents (CA)

	MA (*n* = 58)	CA (*n* = 37)	*p*
One‐finger task			
Right‐hand conditions			
SAME	15.64 (±1.10)	15.73 (±0.60)	.64
CROSSED	14.33 (±1.71)	15.11 (±1.48)	.02*
Left‐hand conditions			
SAME	15.74 (±0.60)	15.95 (±0.22)	.05*
CROSSED	14.02 (±2.23)	14.46 (±1.82)	.31
Two‐finger task			
Right‐hand conditions			
SAME	11.98 (±2.79)	12.27 (±2.38)	.60
CROSSED	9.60 (±3.46)	11.24 (±2.42)	.01*
Left‐hand conditions			
SAME	12.05 (±2.48)	12.65 (±2.27)	.24
CROSSED	9.74 (±3.51)	12.19 (±2.53)	.01**

Note

Data from MA and CA were compared using *Student's* *t* test for the CFLT scores (continuous parametric variable); level of statistical significance *p ≤ .05 ; **p < .01.

Abbreviations: ± *SD*, standard deviation; CA, comparison adolescents; CFLT, Crossed Finger Localization Task; MA, maltreated adolescents.

### 3.3 Neuroimaging: DTI data and association with CM

**Figure 1:**
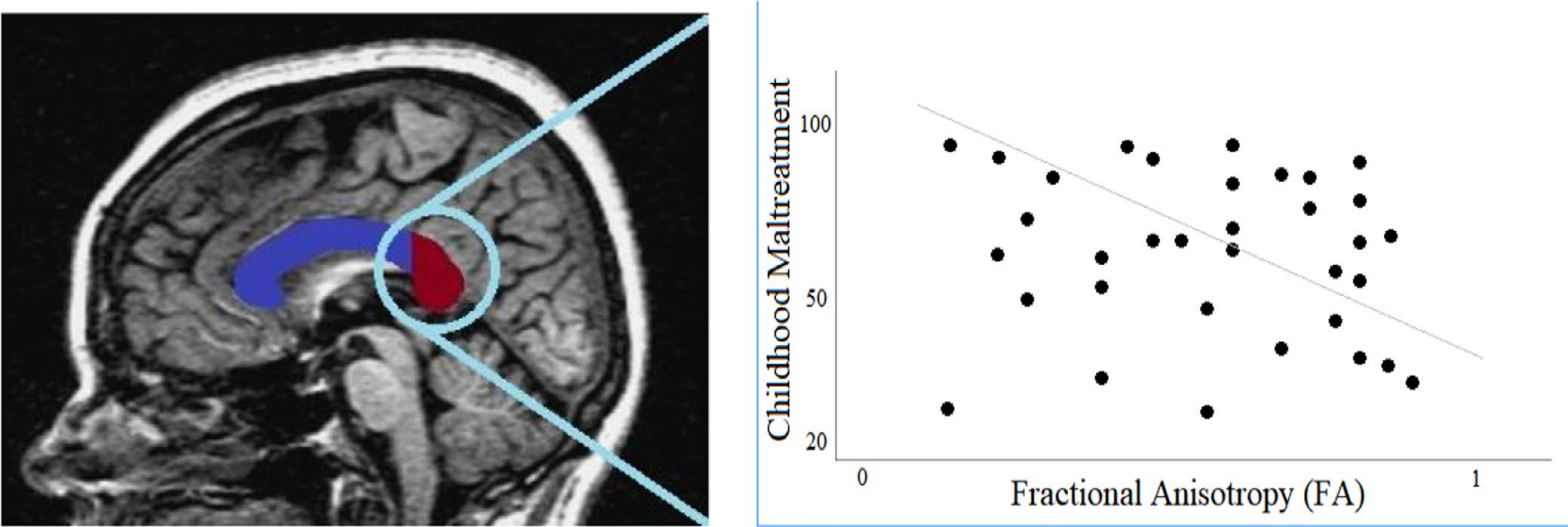
Childhood maltreatment significantly associated with reduced levels of FA in the splenium of the corpus callosum (in red) among maltreated adolescents. Values were considered statistically significant if *p* < .05 corrected; *ρ* = −0.284; *p* = .032; cluster size = 315 voxels, MNI coordinates (26, −55, 13). All statistics were run using TBSS and SPM

Childhood maltreatment (CTQ) was inversely correlated with FA levels in the *splenium* of the CC in the whole group (*p* < .05, FWE; peak MNI coordinates 26, −54, 13; 14 voxels; Table 3; Figure 1). When correlation analyses were carried out separately for the MA and CA groups, the negative correlation between CM (CTQ total score) and FA in the *splenium* of the corpus callosum remained significant only in the MA group (*p* < .05; peak MNI coordinates 26, −54, 13).

**Table 3:** Childhood maltreatment (CM) associated with fractional anisotropy (FA) in maltreated (MA) and comparison adolescents (CA)

CC area	Localization/Voxels	*x*	*y*	*z*	*p* value
Negative correlations (↑ CTQ ↓ FA) Corpus callosum	Splenium (posterior area) 14 voxels	26	−54	13	.01

Note

*p* value corrected for multiple comparisons, FWE_‐corr_ (*p* < .05).

Abbreviations: CA, comparison adolescents; CTQ, Childhood Trauma Questionnaire; FA, fractional anisotropy; MA, maltreated adolescents.

We also found statistically significant direct correlations between FA and CFLT performance in the two‐fingered CROSSED conditions (*p* = .05) among MA and CA.

### 3.4 Child maltreatment and CFLT scores

Childhood maltreatment was negatively correlated with CFLT (*ρ* = −0.25; *p* < .01), meaning that higher levels of CM were associated with lower performance in the two‐fingered CROSSED conditions in both left (*ρ* = −0.21; *p* < .05) and right hands (*ρ* = −0.27; *p* < .01).

### 3.5 Callosal performance and cortisol levels

We also found a negative correlation between CFLT using two fingers in the right hand (*ρ* = −0.27; *p* = .029) and cortisol levels in the whole group.

## 4 DISCUSSION

This study found an association between childhood maltreatment (CM) and deficits in a specific neurocognitive task that requires interhemispheric sensorimotor integration, the CFLT. Also, CM had a direct relationship with altered FA levels in posterior portions (splenium) of the CC in early maltreated adolescents. We also found a significant indirect association between CFLT scores and FA values in the splenium of the CC, meaning that WM microstructure abnormalities might represent a relevant neurobiological substrate underlying the neuropsychological deficits we found on interhemispheric communication. Impairments in the CFLT were more prominent on the CROSSED conditions, thus reinforcing the evidence of an impaired callosal functioning and sensorimotor integration among maltreated adolescents. Finally, the association between CFLT deficits with altered cortisol levels suggests that a chronically altered stress system could have interfered with interhemispheric communication. Further prospective studies would be necessary to better characterize at what extent high cortisol fluctuations and impaired sensorimotor integration among individuals with childhood maltreatment would impact on the neurodevelopmental trajectories, brain microstructure, neurocognition, and emotional regulation later in life.

Our study found an altered WM microstructure in fibers of the splenium of the CC, which is the callosal portion connecting parietotemporal and occipital areas, in association with CFLT, a neurocognitive task that requires processing of sensorimotor information transfer involving the superior and inferior medial temporal (including the posterior cingulate), posterior parietal, and occipital cortices, all of which are interconnected through posterior segments of the CC (Teicher & Samson, 2016). It is noteworthy that the splenium of the corpus callosum shows the greatest degree of growth between 5 and 18 years of age (Luders et al., 2010), thus explaining why adolescents early exposed to maltreatment may be more vulnerable to alterations in these posterior brain associative areas. In this regard, alterations in attention, visuospatial, calculation, memory/learning, and executive functions may be consequences of damage in this area among children exposed to maltreatment (Huang et al., 2012). Considering that the splenium of the CC interconnects the posterior right and left hemispheres, playing an important role on the integration of sensorimotor information, it is also very plausible to hypothesize that this altered WA microstructure could interfere with the normal development of some important social, emotional, and affective aspects. Given the associations of childhood maltreatment with neurocognition, cortisol levels, and WM microstructure, efforts to understand at extent to which such sensorimotor alterations could represent mediators to problems with formation of early affective bonds and later psychopathology are necessary.

Our study has some strengths by implementing a neuropsychiatric translational approach and including multiple measures to characterize abnormalities on different levels. Also, our study is characterized by significant findings when using correlation analyses rather than performing group comparisons. This is in accordance with recent neuroimaging studies in which correlation analyses using continuous variables related to different levels of CM have proved to be more useful than a bicategorical classification (Duarte et al., 2016; Huang et al., 2012; Olson et al., 2019). In fact, some subtle alterations may be evident only in an univariate analysis including more subjects instead of a subgroup analysis. Finally, and perhaps more importantly, there is normally a *continuum* between distinct levels of exposure to early maltreatment in the general population and even individuals from “healthy” control groups may have been exposed to slight and mild levels of early stress situations, which in turn may also correlate with subtle alterations in the callosal microstructure (Paul et al., 2008).

However, our study has a set of limitations. First, the cross‐sectional design and the correlational nature of our investigation do not allow us to infer causality from our results, considering potential temporal causes (including mediation effects, for instance) and the use of a retrospective measure of exposure to early maltreatment, the CTQ, due to possible psychological bias while recalling traumatic events in the childhood. Future longitudinal studies are required to further ascertain the possible link between early maltreatment, WM microstructure, neurocognition, and chronic cortisol dysregulation. Further longitudinal investigations could also provide interesting insights on whether reduced FA in the CC in subjects with a history of CM could be reversed depending on the type of environment they are exposed to or due to resilience factors (Galinowski et al., 2015; Jones et al., 2019; Paul et al., 2008; Sheridan et al., 2012; Teicher & Samson, 2016). Second, the inclusion of boys and girls may have added variability to our DTI data and cortisol results, given that gender may be a relevant factor when considering CM‐related brain functional patterns (Crozier et al., 2014). However, our samples were similar regarding gender distribution and comparative results of CTQ, FA, and cortisol levels revealed no statistically significant differences when comparing boys with girls. In this regard, our data linking cortisol with maltreatment should be interpreted with caution. Third, we evaluated subjects at different ages during adolescence, in a period while several and important brain maturational processes are underway, so our DTI data may have varied across subjects under the influence of subtle biological changes occurring typically during adolescence (Giedd, 2004). However, the samples were similar regarding age. Also, there were no significant differences between MA and CA on a self‐report measure of pubertal status, which has been previously shown to reliably correlate with biological indices of puberty such as hormonal levels in adolescents (Shirtcliff et al., 2009). Fourth, given that the presence of comorbid psychiatric disorders could interfere with brain microstructure and HPA axis controlling cortisol release, we also performed a set of subanalyses to investigate the extent to which our results could be attributable to other psychiatric symptomatology that may also interfere with cortisol levels and the hypothalamic–pituitary–adrenal (HPA) axis (Tables S1–S3). However, our subanalyses showed that maltreated adolescents with psychiatric comorbidities such as conduct, depressive, and attention‐deficit/hyperactivity disorder did not differ from those without psychiatric comorbidities in terms of FA values, thus suggesting that the FA abnormalities we found in the splenium of the CC among MA could not be attributable to current psychopathology, which is consistent with a recent published study (Meinert et al., 2019). Fifth, the use of an 1.5T MRI SIEMENS scanner might have limited the resolution of our neuroimaging findings, so other studies using a 3 Tesla scanner could be better to investigate more subtle alterations in the WM microstructure among maltreated adolescents. Finally, we did not evaluate how different types of maltreatment could be associated with a possible differential impact on WM microstructure, mainly because our restricted sample was composed mostly by early poly‐victimized adolescents.

In sum, the associations between CM, abnormal WM integrity in posterior regions of the CC, and CFLT results add to the literature a direct neuropsychological link between an altered brain interhemispheric connectivity through CC and neurocognitive impairments in adolescents early exposed to maltreatment. Further prospective studies are necessary to investigate the extent to which these sensorimotor and interhemispheric functional abnormalities and cortisol dysregulation might negatively impact on the integration of other cognitive and affective aspects, thus predisposing early victims of maltreatment to later psychopathological symptoms such as substance use, depression, and anxiety.

## CONFLICT OF INTEREST

The authors declare that there is no conflict of interest.

## AUTHOR CONTRIBUTIONS

Geraldo F. Busatto, Sandra Scivoletto, and Paulo J. Cunha contributed to study concept and design. Paulo J. Cunha, Sandra Scivoletto Geraldo F. Busatto, Paula A. de Oliveira, Fabio L. S. Duran, Tiffany M. Chaim‐Avancini., Ana Luiza V. Milioni, Mariella Ometto, Paula Squarzoni, Pedro P. Santos, and Sheila C. Caetano analyzed and interpreted the data. Paulo J. Cunha drafted the manuscript. Geraldo F. Busatto, Sandra Scivoletto, Tiffany M. Chaim‐Avancini, Paula A. de Oliveira, and Sheila C. Caetano critically revised the manuscript for important intellectual content. Paulo J. Cunha and Fabio L. S. Duran performed statistical analysis. Geraldo F. Busatto, Sandra Scivoletto, and Paulo J. Cunha obtained funding. Geraldo F. Busatto and Sandra Scivoletto underwent study supervision.

## ETHICAL APPROVAL

IRB approval for this study was obtained from the Hospital das Clinicas Research Ethics Committee of the University of São Paulo, School of Medicine, and all procedures were implemented according to the Declaration of Helsinki. All the adolescents from MA, CA, and also their legal guardians gave their informed consent prior to their inclusion in the study. Finally, all the adolescents and their parents or legal guardians were reimbursed for their basic financial costs and transportation during the evaluation process.

### Peer Review

The peer review history for this article is available at https://publons.com/publon/10.1002/brb3.2009.

## Supporting information

TableS1‐S3

## Data Availability

The data that support the findings of this study are available from the corresponding author upon reasonable request.
