# Supplementary material for: Callosal abnormalities, altered cortisol levels, and neurocognitive deficits associated with early maltreatment among adolescents: A voxel‐based diffusion‐tensor imaging study
Source: Brain Behav. 2021 Jan 15;11(3):e02009. doi: 10.1002/brb3.2009 (PMC7994704; doi:10.1002/brb3.2009)
Supplement: Supplementary file 1 — TableS1‐S3 [file BRB3-11-e02009-s001.docx]

**Table S1.** Comparative sub-analyses among maltreated adolescents (MA) with Depressive Disorders and without Depressive Disorders.

| **Fractional Anisotropy (FA)** | **Depressive Disorders**  (n= 18) | **Without Depressive Disorders**  (n=40) | p |
| --- | --- | --- | --- |
|  |  |  |  |
| Corpus Callosum (CC) | 0.71 (0.12) | 0.74 (0.07) | .365 |
|  |  |  |  |

**Notes:** FA: Fractional Anisotropy; FA values are presented in means and standard deviations: M (±SD); FA values from MA with Depressive Disorders compared with MA without Depressive Disorders were compared using *Student* T-test; level of statistical significance *p≤.05 ; ** p<.01.

**Table S2.** Comparative sub-analyses among maltreated adolescents (MA) with Conduct Disorders and without Conduct Disorders.

| **Fractional Anisotropy (FA)** | **Conduct Disorders**  (n= 47) | **Without Conduct Disorders**  (n=11) | p |
| --- | --- | --- | --- |
|  |  |  |  |
| Corpus Callosum (CC) | 0.71 (0.09) | 0.73 (0.17) | .606 |
|  |  |  |  |

**Notes:** FA: Fractional Anisotropy; FA values are presented in means and standard deviations: M (±SD); FA values from MA with Conduct Disorders compared with MA without Conduct Disorders were compared using *Student* T-test; level of statistical significance *p≤.05 ; ** p<.01.

**Table S3.** Comparative sub-analyses among maltreated adolescents (MA) with Attention-Deficit Hyperactivity Disorders (ADHD) and without ADHD.

| **Fractional Anisotropy (FA)** | **ADHD**  (n= 20) | **Without ADHD**  (n=38) | p |
| --- | --- | --- | --- |
|  |  |  |  |
| Corpus Callosum (CC) | 0.70 (0.13) | 0.72 (0.09) | .520 |
|  |  |  |  |

**Notes:** FA: Fractional Anisotropy; FA values are presented in means and standard deviations: M (±SD); FA values from MA with ADHD compared with MA without ADHD were compared using *Student* T-test; level of statistical significance *p≤.05 ; ** p<.01.
